# Effects of Intra-articular Platelet Rich Plasma on Cartilage Thickness, Clinical and Functional Outcomes in Knee Osteoarthritis

**DOI:** 10.7759/cureus.32256

**Published:** 2022-12-06

**Authors:** Deepthi S Johnson, Nitish Dhiman, Suman Badhal, Ranjan Wadhwa

**Affiliations:** 1 Physical Medicine and Rehabilitation, Vardhman Mahavir Medical College and Safdarjung Hospital, New Delhi, IND

**Keywords:** ultrasonography, platelet-rich plasma/ prp, knee osteo-arthritis, knee osteoarthritis outcome score, visual analogue scale, cartilage thickness

## Abstract

Background: Osteoarthritis of the knee is one of the most common degenerative diseases and the fourth leading cause of years lived with disability at the global level. This study assessed the efficacy of platelet-rich plasma (PRP) in osteoarthritis of knees as to changes in cartilage thickness and clinical and functional outcomes.

Methods: Thirty participants with Kellgren-Lawrence grade two and grade three osteoarthritis knee who satisfied the inclusion and exclusion criteria were enrolled in this prospective interventional study after taking written informed consent. Each participant received three doses of two ml intraarticular platelet-rich plasma at an interval of seven days. Clinical assessment was determined using the Visual Analogue Scale (VAS) and Knee Osteoarthritis Outcome Score (KOOS) on Day 0, Day 90, and Day 180. Cartilage thickness (femoral and trochlear cartilage) was measured pre (Day 0) and post-PRP (Day 180) under ultrasound guidance.

Results: The mean VAS score for pain was 7.4 before treatment which changed to 5.3 (p= <0.0001) on Day 90 and 3.37 (p= <0.0001) on Day 180 post-PRP. The mean total KOOS was 19.16 ± 10.73 before treatment which improved to 37.42 ± 9.88 (p= <0.0001) and 49.98 ± 8.82 (p= <0.0001) at 90 days, and 180 days post-injection, respectively. The mean cartilage thickness (femoral and trochlear cartilage) improved from baseline (day 0) to final follow-up on day 180, which was statistically significant and implied cartilage repair following PRP administration.

Conclusion: This study supports the efficacy of PRP in the management of osteoarthritis knee by improvement in pain, joint stiffness, and activities of daily living, as well as aids in the repair and regeneration of articular cartilage.

## Introduction

Osteoarthritis (OA) is the fourth leading cause of years lived with disability at the global level. Increased longevity teamed with the epidemic of obesity and the resultant motivation to exercise, often through sports, the burden and prevalence of OA are expected to grow further [1,2]. Clinically, OA presents with recurring episodes of pain, particularly after prolonged activity and weight bearing that decreases with rest, stiffness felt after inactivity (gel phenomenon), progressive limitation of movement, and synovitis with effusion [3].

American College of Rheumatology (ACR) recommends various pharmacological and non-pharmacological treatment modalities for the management of knee OA [4]. Weight reduction, joint offloading (knee braces, cane, or walker), exercises, Tai Chi, and therapeutic modalities (thermal treatments) are a few of the non-pharmacological therapies [5,6]. Pharmacotherapy chiefly includes acetaminophen, non-steroidal anti-inflammatory drugs (NSAIDS) oral as well as topical, intra-articular corticosteroids, opioids, and topical capsaicin. Surgical management includes arthroscopic debridement, osteotomy of the proximal tibia or distal femur, uni-compartmental knee replacement, total knee replacement, etc. are mostly reserved for more severely disabled patients who have failed conservative management [4,7,8].

Conservative treatments increase the quality of life of patients, especially in the early phase. Current researchers are investigating new methods of stimulating repair or replacing damaged cartilage [9]. Platelet Rich Plasma (PRP) has the function of chondrogenesis, proliferation of fibroblasts in vitro, regulation of metalloproteinases, collagen synthesis, and stimulation of synovial fibroblast to produce hyaluronic acid that repairs the damaged articular cartilage [10].

PRP obtained from centrifuging the autologous venous blood contains a high concentration of platelets (2-5 times of whole blood) in a small plasma volume. The PRP is made to improve clinical and structural outcomes by delivering a high concentration of growth factors that mediate cartilage healing and remodeling [11].

There is disagreement regarding the clinical use of intra-articular PRP for knee OA. American College of Rheumatology and Osteoarthritis Research Society International treatment guidelines have advised against the use of PRP for the treatment of knee OA due to low-quality evidence [12,13]. In the previous studies, it has been observed that there is clinical improvement in terms of pain and quality of life, and cartilage thickness, assessed by ultrasonography (USG) or magnetic resonance imaging following intra-articular PRP injection indicating its chondroprotective action [14,15]. Hence based on the previous studies, we hypothesized that there is improvement in cartilage thickness and clinical outcome in knee OA following PRP injection.

The aim was to study the efficacy of intra-articular autologous PRP in osteoarthritis knees using the clinical outcome Visual Analogue Scale (VAS) and Knee Osteoarthritis Outcome Score (KOOS) also USG evaluation for cartilage thickness. The secondary objective was to correlate cartilage thickness change with the clinical outcome.

## Materials and methods

**Figure 1:**
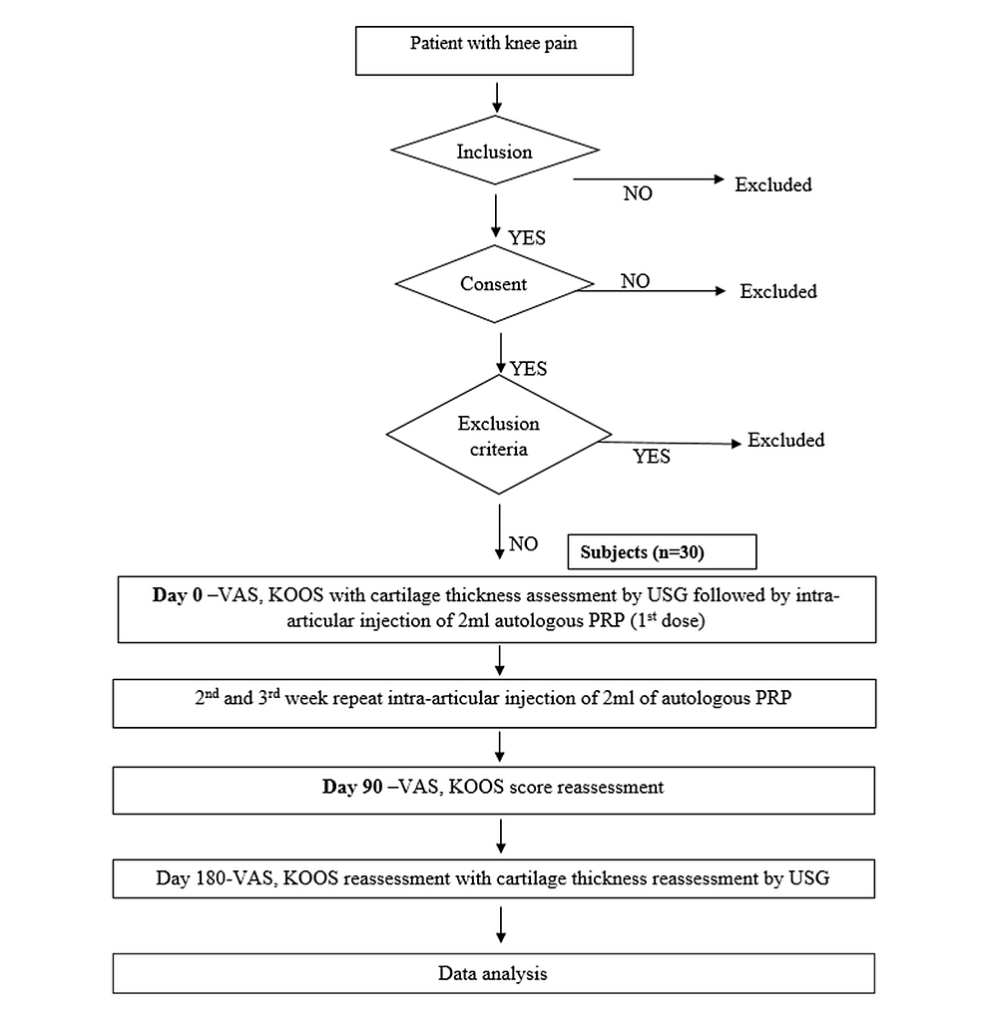
Flowchart showing the progression of the study from screening to data analysis.

This prospective interventional study was conducted in the Department of Physical Medicine and Rehabilitation of a tertiary care hospital in India from October 2018 to July 2020. The study was undertaken after the approval of the Institutional Ethical Committee (IEC/VMMC/SJH/Thesis/October/2018-50), and the study was registered at CTRI.nic.in (CTRI/2020/03/023685). The sample size of the study was 30 which was calculated based on the prevalence (3.28%) of knee OA in Delhi [1]. Patients with primary knee OA of tibiofemoral joint as defined by the American College of Rheumatology clinical criteria with radiological Kellgren Lawrence grade two and three belonging to 50-75 years of age, irrespective of gender, were included in the study [16,17]. Those with inflammatory arthritis, bleeding disorders, thrombocytopenia, intra-articular treatment with any product, previous arthroscopic procedure or knee surgery, and any use of anticoagulants or non-steroidal anti-inflammatory drugs within the previous seven days were excluded. All participants provided written informed consent after the procedure was explained to them (Figure 1).

NSAIDs (non-steroidal anti-inflammatory drugs) or other analgesic medications were not allowed throughout the study period, except acetaminophen (maximum dose of 2g/day) if the pain was unbearable, and the number of tablets/days was noted. Taking all aseptic precautions 6 mL of blood was collected in two sterile sodium citrate-coated vacutainer tubes and was centrifuged at the rate of 2500 rotations per minute (rpm) for 5 minutes in a centrifuge machine REMI CENTRIFUGE R-8C DX (REMI ELEKTROTECHNIK LTD, Vasai, India). PRP was prepared at the department of Physical Medicine and Rehabilitation (PMR). After centrifugation the whole blood is separated into three layers: the upper layer mainly of plasma and a few WBCs (white blood cells), the buffy coat layer that is rich in platelets and WBCs, and the third layer of RBCs (red blood cell). These upper two fractions were used for injections. The fraction obtained was taken to the blood bank and subjected to complete blood counts in the initial few cases to confirm a standard platelet count in PRP of two to five times the original count in the blood.

All patients enrolled in the study received three consecutive doses of two ml PRP intra-articular injection in the affected knee at an interval of one week by a Physical Medicine and Rehabilitation (PMR) specialist through a lateral approach using aseptic precautions. Each patient was evaluated on day 0 and then on days 90 and 180. Post-procedure protocol followed was an isometric strengthening exercise of the quadriceps and hamstring muscles and general precautions in Activities of Daily Living (ADL).

**Figure 2:**
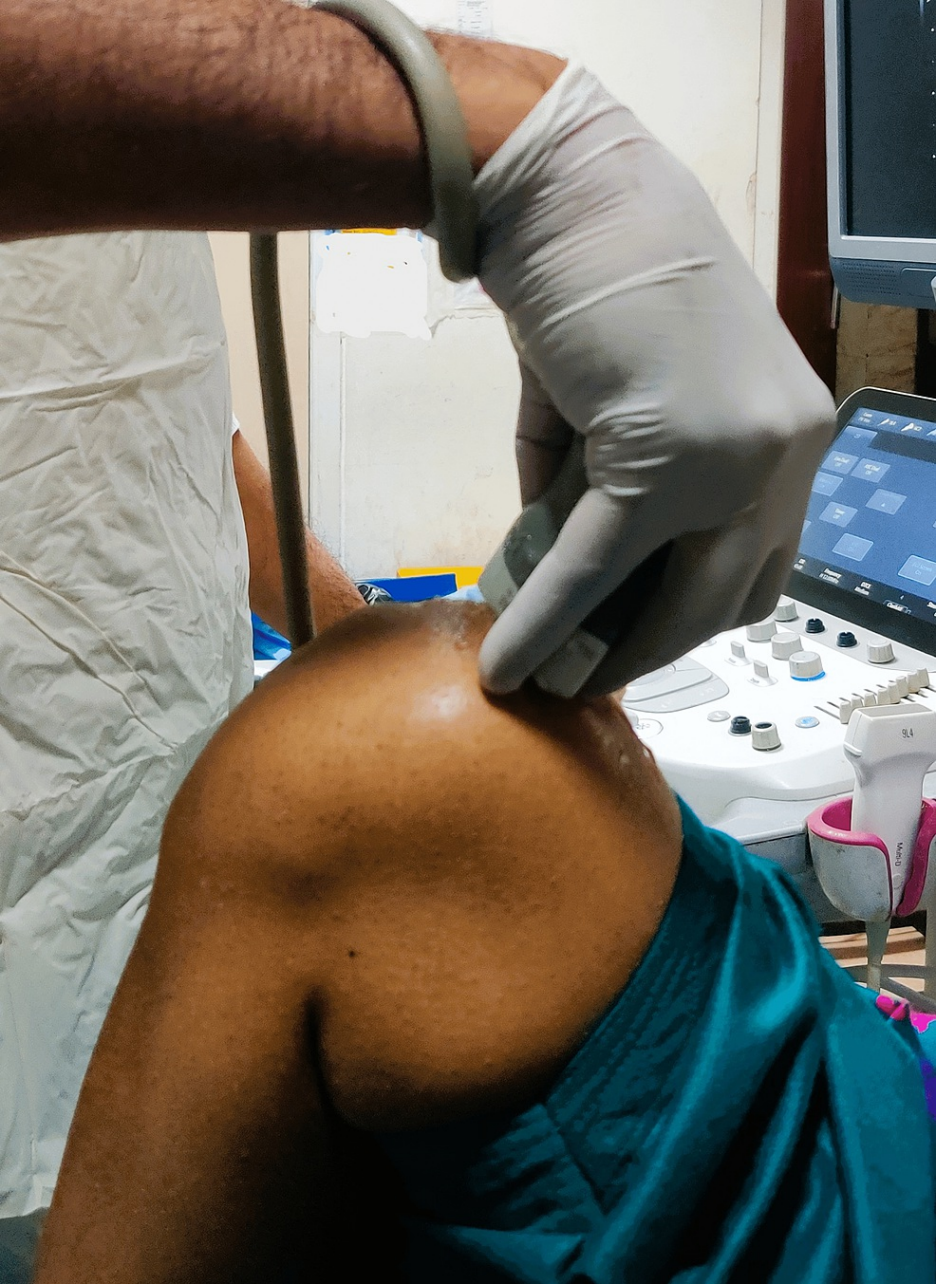
Image showing the placement of the ultrasound probe and patient positioning while assessing femoral cartilage thickness. (Permission taken for the photograph)

**Figure 3:**
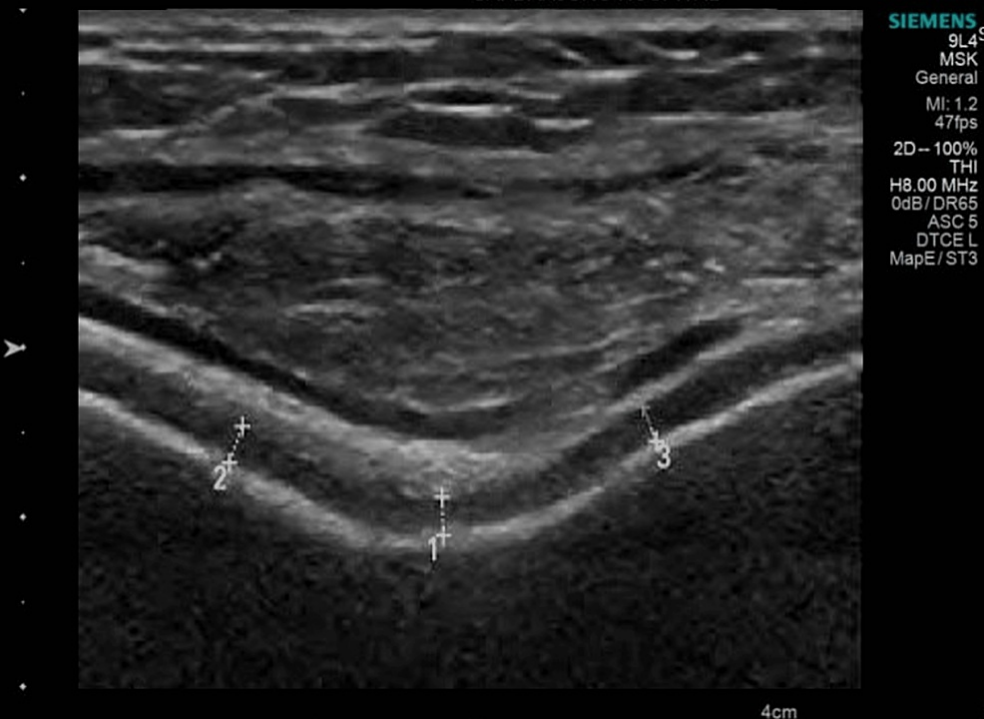
Ultrasonogram of femoral trochlear notch cartilage. 1- Middle trochlear notch; 2- Medial trochlear notch; 3- Lateral trochlear notch

Patients were clinically evaluated using VAS and KOOS on Day 0 (pre-PRP), Day 90, and Day 180 (post-PRP). Cartilage thickness assessment on USG was done by a senior radiologist using Siemens Accuson 3000 (frequency 5-18 Megahertz) (Siemens Medical Solutions, CA, USA) at the trochlear notch, medial and lateral femoral condyles in each knee on day 0 and day 180. The transducer was positioned in the axial plane on the suprapatellar region. All patients were placed in the supine position with maximum knee flexion. Cartilage thickness was measured from the thin hyper-echoic line at the soft tissue cartilage interface to the hyper-echoic line at the cartilage-bone interface [18] (Figures 2, 3).

After getting the clinical evaluation scores and cartilage thickness pre-and post-PRP, data was compiled in an MS EXCEL spreadsheet, and analysis was done using Statistical Package for Social Sciences (SPSS) version 21.0 (IBM Corp., Armonk, NY). Categorical variables were presented in number and percentage (%) and continuous variables were presented as mean ± SD and median. Quantitative variables were compared using the independent t-test/Mann-Whitney Test (when the data sets were not normally distributed) between the two groups and paired T-test/Wilcoxon signed-rank test was used for comparison between pre and post. The correlation of cartilage thickness with clinical outcome was done by statistical analysis using the Spearman rank correlation coefficient/ Pearson correlation coefficient. A p-value of <0.05 was considered statistically significant.

## Results

A total of 30 patients were injected with PRP. Altogether, 20 females and 10 males were included in the study. The mean age of the group was 55.53 ± 4.05 years. 80% of the patients were of K-L grade three knee OA and 20% were grade two knee OA. 66% of the patients had a body mass index (BMI) of more than 25 (Table 1). 

**Table 1:** Basic characteristics of patients included in the study.

BASIC CHARACTERISTICS	FREQUENCY	PERCENTAGE
Age (in years)		
<55	14	46.67%
>55	16	53.33%
Gender		
Male	20	66.67%
Female	10	33.33%
Kellgren Lawrence Grading		
Grade two	6	20.00%
Grade three	24	80.00%
Body Mass Index (kg/m^2^)		
18.5-22.9	4	13.33%
23-24.9	6	20.00%
>25	20	66.66%

**Figure 4:**
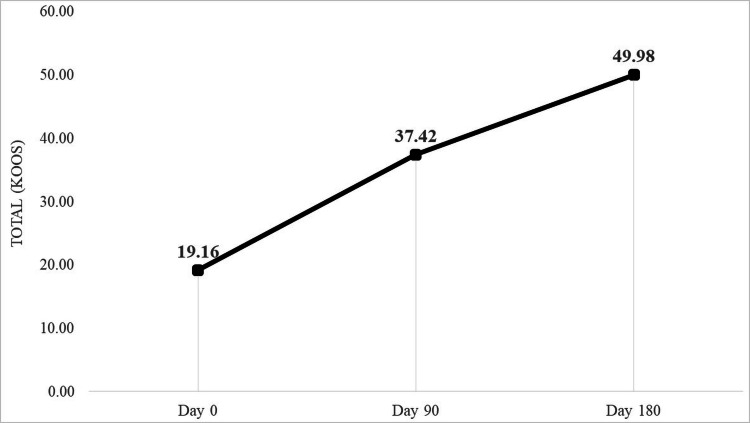
KOOS assessment showing improvement in the scores pre (day 0) and post (day 90 and day 180) intervention. Knee Osteoarthritis Outcome Score (KOOS)

**Figure 5:**
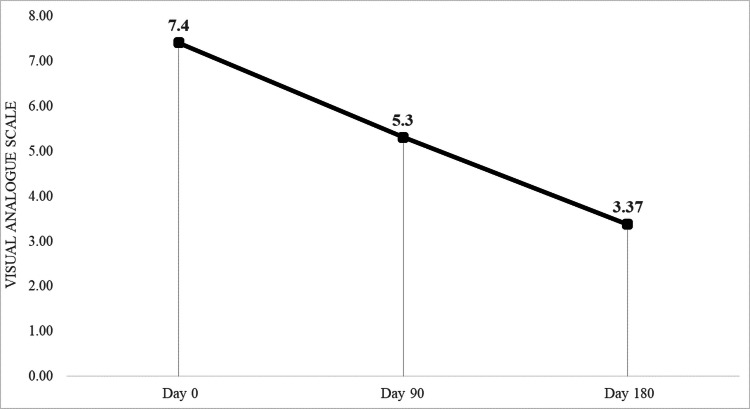
VAS assessment showing improvement in the scores pre (day 0) and post (day 90 and day 180) intervention. Visual Analogue Scale (VAS)

Assessment of pain was done using VAS and KOOS pain scores. Results showed significant improvement (p<0.0001) at 90 days and 180 days follow-up compared with the initial scores. A similar observation was seen with symptom scores, activities of daily living, Sports/recreational scores, and quality of living. The total KOOS and VAS scores in our study showed statistically significant improvement (Table 2; Figures 4, 5).

**Table 2:** VAS and KOOS assessment pre-and post-PRP. Activities of Daily Living (ADL); Quality of life (QOL); Visual Analogue Scale (VAS); Knee Osteoarthritis Outcome Score (KOOS); Platelet-rich plasma (PRP)

		Pre PRP	Post PRP	
		Day 0	Day 90	Day 180
VAS	Mean± SD	7.4 ± 1	5.3 ± 0.95	3.37 ± 0.76
	p-value		<0.0001	<0.0001
Pain KOOS	Mean± SD	18.8 ± 13.08	37.03 ± 11.52	51.11 ± 11.75
	p-value		<0.0001	<0.0001
Symptom KOOS	Mean± SD	17.02 ± 11.81	37.02 ± 10.03	53.09 ± 9.55
	p-value		<0.0001	<0.0001
ADL KOOS	Mean± SD	22.5 ± 13.3	39.31 ± 10.73	52.01 ± 10.76
	p-value		<0.0001	<0.0001
Sports & Recreation KOOS	Mean± SD	10.83 ± 12.18	28 ± 13.68	40 ± 13.58
	p-value		<0.0001	<0.0001
QOL KOOS	Mean± SD	20 ± 12.54	35.63 ± 10.78	45.83 ± 10.29
	p-value		<0.0001	<0.0001
Total KOOS	Mean± SD	19.16 ± 10.73	37.42 ± 9.88	49.98 ± 8.82
	p-value		<0.0001	<0.0001

Cartilage thickness of the knee joint (trochlear and femoral condylar) was measured using USG pre (day 0) and post (day 180) PRP injection and correlated the cartilage thickness changes at baseline with six months post-injection follow-up. The USG findings showed significant improvement in the cartilage thickness of all variables (Table 3).

**Table 3:** Cartilage thickness assessment by ultrasonography on baseline day 0 and day 180 (post-intervention). Millimeter (mm); Ultrasonography (USG)

Cartilage Thickness in mm (USG)		Day 0	Day 180
Middle Trochlear Notch	Mean ± SD	2.43 ± 0.57	2.66 ± 0.54
	p-value	0.002	
Medial Trochlear notch	Mean ± SD	2.15 ± 0.6	2.4 ± 0.42
	p-value	0.0008	
Lateral Trochlear notch	Mean ± SD	1.99 ± 0.54	2.34 ± 0.46
	p-value	0.0002	
Medial Femoral Condyle	Mean ± SD	0.8 ± 0.48	1.56 ± 0.38
	p-value	<0.0001	
Lateral Femoral Condyle	Mean ± SD	1.13 ± 0.58	1.98 ± 1.38
	p-value	<0.0001	

Further, no correlation was observed between the change in cartilage thickness with VAS and KOOS from day 0 to day 180 (Table 4).

**Table 4:** Correlation of change in Cartilage Thickness with VAS and KOOS (Day 0 to Day 180). Visual Analogue Scale (VAS); Knee Osteoarthritis Outcome Score (KOOS)

	Cartilage Thickness Change	Lateral Femoral Condyle	Lateral Trochlear Notch	Medial Femoral Condyle	Medial Trochlear Notch	Middle Trochlear Notch
KOOS	Correlation Coefficient	0	-0.315	0.015	-0.035	0.111
	p-value	0.9991	0.0898	0.937	0.8536	0.5601
VAS	Correlation Coefficient	-0.118	0.133	-0.056	-0.02	-0.132
	p-value	0.5335	0.483	0.7691	0.9169	0.4852

## Discussion

Knee OA, a degenerative joint disease, is one of the leading causes of disability worldwide. Therefore, the treatment should be aimed at relieving pain, improving function, and limiting disabilities [1]. The objective of our study was to assess the effectiveness of intra-articular PRP injections in patients with grade two and three knee OA in terms of pain, functional status, and cartilage regeneration.

Pain is the characteristic symptom of OA and is often the only symptom the patient seeks medical attention for [3]. In this study, the maximum number of patients enrolled was for the complaints of knee pain followed by joint stiffness. Assessment of pain was measured using VAS and KOOS pain scores. Results showed that; there was a significant improvement in pain (p<0.0001) at 90 days and 180 days follow-up. In a study by Filardo et al., significant improvements in pain scores were seen in patients of knee OA who received three PRP injections at six- and 12-month follow-ups [19]. Meta-analyses found that the PRP injections were more effective than the placebo in providing overall clinical improvement up to twelve months after treatment [20,21].

Likewise, substantial improvement was observed in KOOS symptoms, activities of daily living, sports/recreational, quality of life scores, and total KOOS. This observation was in accordance with the randomized control study by Guvendi et al. where single-dose PRP and corticosteroids were given in patients with grade two and three knee OA and pain, symptoms, activities of daily living, and quality of life were significantly improved in the PRP group compared to the corticosteroid group at second-and sixth-month follow-ups [22]. Two separate meta-analyses reported that the functional improvement in knee OA patients treated with PRP was more than in hyaluronic acid [2,23].

The USG findings showed significant improvement in the cartilage thickness of all variables (trochlear and femoral condylar) on day 180 post-PRP injection. The baseline cartilage thickness was correlated with the baseline KOOS score and VAS score and no significant correlation was observed. Similarly, the improvement in cartilage thickness was correlated with the improvement in KOOS score and VAS score, and no correlation was observed for the same. It implies that the severity of the clinical symptoms has no significant correlation with the amount of cartilage degraded nor there is any significant correlation with the amount of cartilage regenerated with the reduction in symptoms. It may be attributed to the effect of PRP in synovitis and increasing hyaluronic acid production. This was in accordance with the study of Halpern et al. who concluded the chondroprotective action of PRP in OA [15]. Hart et al. evaluated PRP response in 50 patients with knee OA by MRI before and one year after the injection. Cartilage thickness remained unchanged in 94% of cases, but a slight increase (less than one mm) was recorded in three cases (6%) [24]. A similar study was conducted by Sampson et al. who evaluated the effect of PRP in 14 patients with knee OA using the KOOS and Brittberg-Peterson VAS scores, did the follow-up for 52 weeks (one year), and found significant clinical improvement. In addition to clinical response assessment, a joint ultrasound was done to measure the articular cartilage thickness one year after PRP injection. Though the results showed no significant increase in the thickness of the articular cartilage, six of the 13 patients demonstrated increased femoral articular cartilage at the lateral condyle, medial condyle, and intercondylar notch [14].

While doing this study, one of the main limitations observed was that there is no standardization regarding the speed, duration, and the number of spins needed in the preparation of PRP nor about the layer which has to be exactly removed from the precipitate. PRP is prepared by a process known as differential centrifugation. In this method, acceleration force is regulated to sediment some cellular constituents of blood, based on the difference in specific gravity, and leave others in suspension.

There are several methods to prepare PRP, namely the single spin method and the double spin method. The usage of two spins versus one spin is controversial, even though a second spin will certainly concentrate the platelets further [9,19]. Few studies compared different methods of preparation of PRP and found that the single spin method provides a platelet concentration of 2.19 of the baseline while the double spin method gives a platelet concentration as high as 3.36 to 5 times the baseline [21,25]. Graziani et al. suggested that the optimal concentration of PRP was 2.27 times the baseline value, and above this, there may be an inhibitory effect on healing [26]. In this study, the target platelet concentration to reach was two-three times that of whole blood, and it could be achieved with a single centrifuge method.

Different PRP preparations depending on the leukocyte and fibrin content: pure PRP, leukocyte-rich PRP, pure platelet-rich fibrin, and leukocyte and platelet-rich fibrin have been described. The superiority of one PRP formulation over another is questionable due to a lack of consistency. The one used in our study was leucocyte-rich PRP as we did not separate leucocytes from PRP. This might be beneficial, as leukocyte-containing PRP could have some role in preventing injection site infection and in prolonging growth factor releasing time [9,27,28].

Platelet activation is an important step that influences the delivery of growth factors by degranulation which can be done by bringing it in contact with calcium chloride/ thrombin/ collagen. In our study, we preferred in vivo collagen activation as it leads to a slower and more sustained release of growth factors [29]. We gave three consecutive doses of PRP in a gap of one week because the half-life of a platelet is seven days and repeated doses are probably needed to sustain the gain achieved. Subramanyam et al. in their study observed that intra-articular administration of three doses of PRP yields superior outcomes to single and double doses at the end of one year [30].

As PRP is an autologous preparation, it is safe, free from concerns over blood-transmittable diseases, and affordable. Although some complications like pain and swelling were observed in a few studies, no such serious adverse events were observed in our follow-up period.

The findings of our study demonstrated the efficacy of PRP in the treatment of osteoarthritis knee and are supported by previously reported findings by various researchers. The radiologist who measured the cartilage thickness was blinded regarding pre or post-PRP measurement, other investigators were not blinded. However, the shortcomings we acknowledge are the absence of a comparative control group and a small cohort of 30 patients.

## Conclusions

Intra-articular administration of PRP in knee OA aids in the regeneration and repair of articular cartilage, improvement in pain, symptoms, joint stiffness, and quality of life, and reduces the disability associated with it thereby confirming its effectiveness. Hence, PRP could be considered a promising and cost-effective treatment option in the management of knee OA. However, a study involving a larger sample size to ascertain its treatment efficacy is warranted to authenticate the findings of the present study.
